# Seized by Blood: A Case of Neurogenic Pulmonary Edema Presenting As Diffuse Alveolar Hemorrhage

**DOI:** 10.7759/cureus.67308

**Published:** 2024-08-20

**Authors:** Kaitlyn N Romero, Anvit D Reddy, Oshin Rai, Abhinav Karan, Azeem Rathore, Rafik Jacob

**Affiliations:** 1 Internal Medicine, University of Florida College of Medicine – Jacksonville, Jacksonville, USA; 2 Internal medicine, University of Florida College of Medicine – Jacksonville, Jacksonville, USA; 3 Program for Adults with Intellectual and Developmental Disabilities, University of Florida College of Medicine – Jacksonville, Jacksonville, USA

**Keywords:** neurogenic pulmonary edema, dah, hemoptysis, seizure, non-cardiogenic pulmonary edema

## Abstract

Neurogenic pulmonary edema (NPE) is a condition that is characterized by acute onset respiratory distress that uncommonly can cause diffuse alveolar hemorrhage (DAH). Our case is based on a 41-year-old female with a past medical history of seizure disorder who presented for shortness of breath and hemoptysis after a seizure. A computed tomography (CT) scan of the lungs revealed patchy ground glass subpleural airspace opacities bilaterally with increased secondary pulmonary lobule interstitial thickening. With concerns for DAH, a bronchoscopy was performed and revealed sequentially bloody aliquots. Infectious and autoimmune testing was negative. This case highlights a rare form of DAH arising from NPE.

## Introduction

Neurogenic pulmonary edema (NPE) is characterized by respiratory distress secondary to an acute onset of pulmonary edema not due to cardiac or pulmonary causes [1]. Patients often present with dyspnea, tachypnea, cough, frothy sputum, and less commonly hemoptysis [1,2]. The current pathogenesis of NPE is not fully understood, but it can have fatal outcomes in patients resulting in intubation and requiring intensive level of care [3]. NPE is diagnosed due to an acute onset of respiratory distress, typically 30-60 minutes following a central nervous system event, which includes seizures, cerebral infarcts, subdural hemorrhages, epidural hemorrhages, and many other neurological events that affect the cerebral parenchyma. Diagnosis is typically made with imaging significant for diffuse bilateral or unilateral alveolar opacities and infiltrates without cardiomegaly and with the exclusion of other etiologies such as heart failure and infection [1]. Treatment of NPE includes supportive care such as vasoactive compounds, diuretics, supplemental oxygen, and even mechanical ventilation in severe cases. NPE can have a rapid resolution in patients when the etiology is known and inciting events are prevented [1,3].

Diffuse alveolar hemorrhage (DAH) typically presents with hemoptysis in a patient found to have anemia and radiographic findings of diffuse pulmonary infiltrates [4]. DAH can lead to significant respiratory distress and result in mechanical ventilation due to alveoli being filled with blood and impaired oxygenation [4,5]. There are multiple autoimmune, infectious, and toxic etiologies known to cause DAH, but there are fewer common causes that include NPE [4,5]. DAH is a treatable condition but can be life-threatening when the cause is unknown and directed therapy is unobtainable [4].

This case presentation focuses on a case of a patient with the rapid resolution of hemoptysis in the setting of DAH, with an etiology most consistent with NPE.

## Case presentation

**Figure 1:**
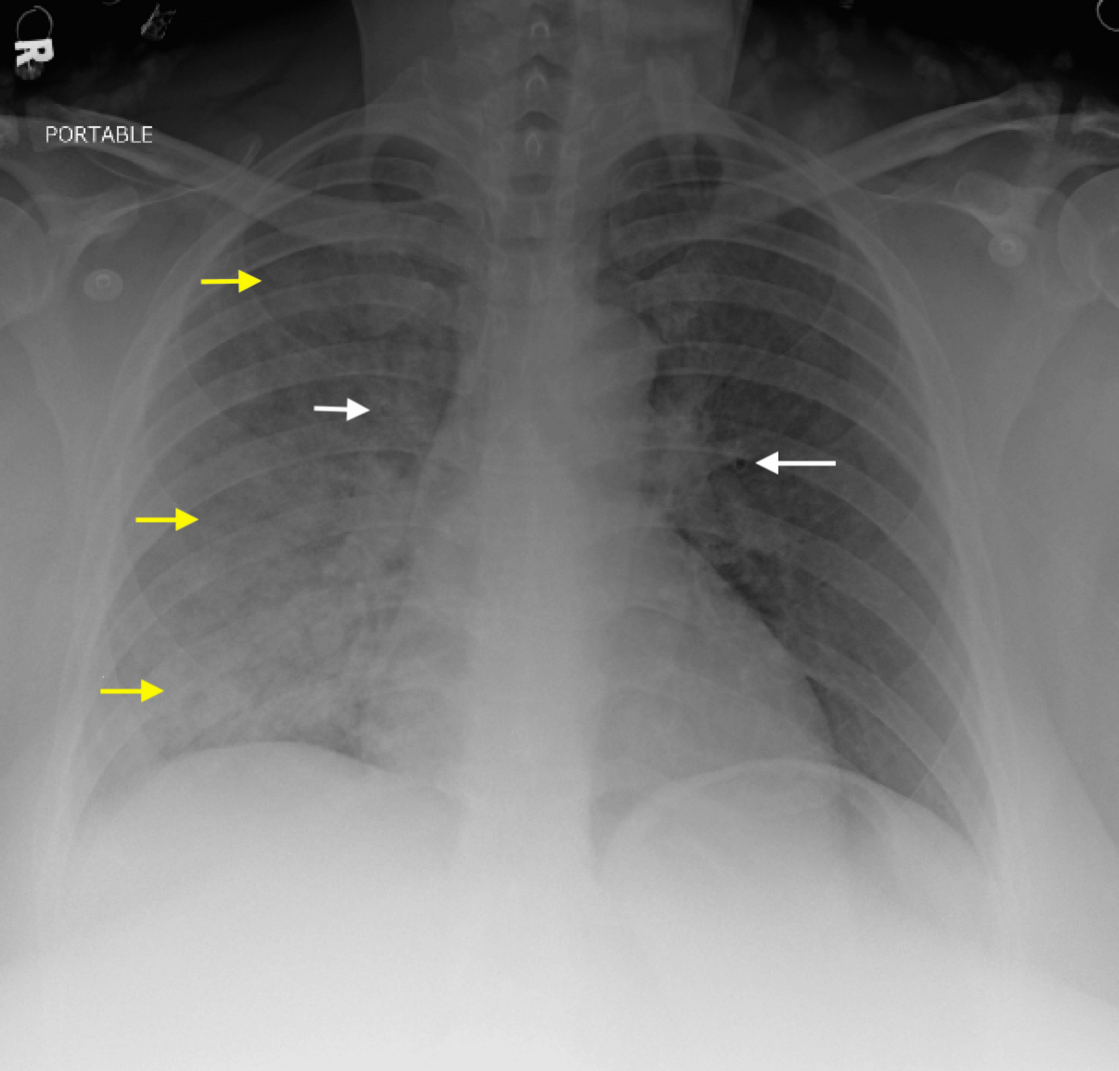
Chest X-ray: Bilateral alveolar opacities, with predominance in the right lung Yellow arrows: Pulmonary alveolar opacity consistent with pulmonary edema, white arrows: peribronchial thickening consistent with pulmonary edema

A 41-year-old female with a past medical history of a seizure disorder presented for hemoptysis following a seizure. The patient had a single, generalized tonic-clonic seizure that lasted approximately three minutes. Upon presentation, the patient had active, large-volume hemoptysis with no source of oropharyngeal bleeding seen on exam. Her vitals on arrival were a blood pressure of 142/46 mmHg, a heart rate of 87 beats per minute, 26 breaths per minute, and 98% oxygen saturation on room air. She later became hypoxic and required four liters of oxygen to maintain an oxygen saturation of 92%. Physical exam was significant for rales throughout the right lung fields. A chest radiograph (Figure 1) demonstrated an alveolar pulmonary opacity, filling the right lung almost entirely.

Laboratory evaluation revealed a D-dimer elevated to 10,700 ng/dL, tropinemia of 18 ng/dL with delta of 7 ng/dL, and leukocytosis of 24 thou/cumm. Renal function was within normal limits (1.01 mg/dL) with no other electrolyte abnormalities. A creatine kinase and lactic acid were not obtained. N-terminal pro-brain natriuretic peptide (NT-proBNP) was mildly elevated to 233 pg/mL. The hemoglobin was stable compared to prior admissions at 13 G/dL and coagulation studies were within normal limits. A respiratory viral panel was negative.

**Figure 2:**
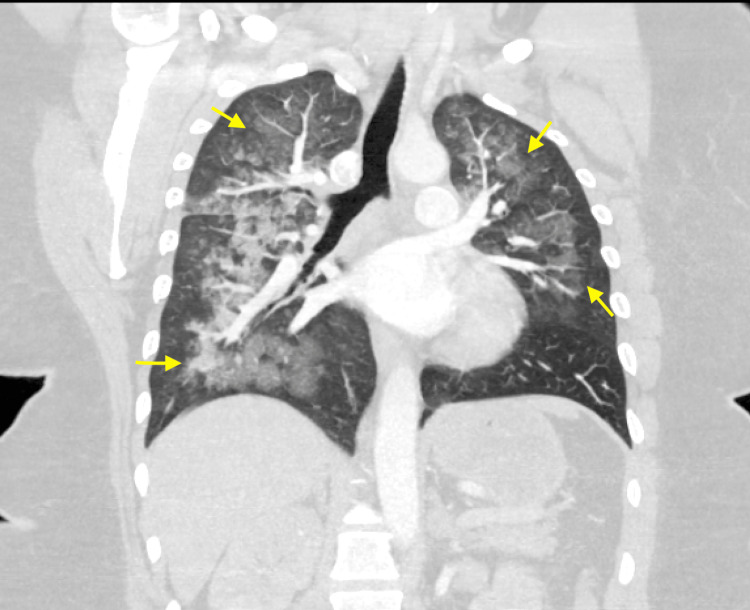
Coronal view of the bilateral lung fields demonstrating diffuse patchy ground glass opacities Yellow arrows: Outlining the extent of alveolar infiltrates

A computed tomography (CT) scan of the lungs (Figure 2) revealed patchy ground glass subpleural airspace opacities seen within the bilateral lung zones with increased secondary pulmonary lobule interstitial thickening. There were no filling defects of the pulmonary arteries, pleural effusions, pneumothorax, or hemothorax seen.

**Figure 3:**
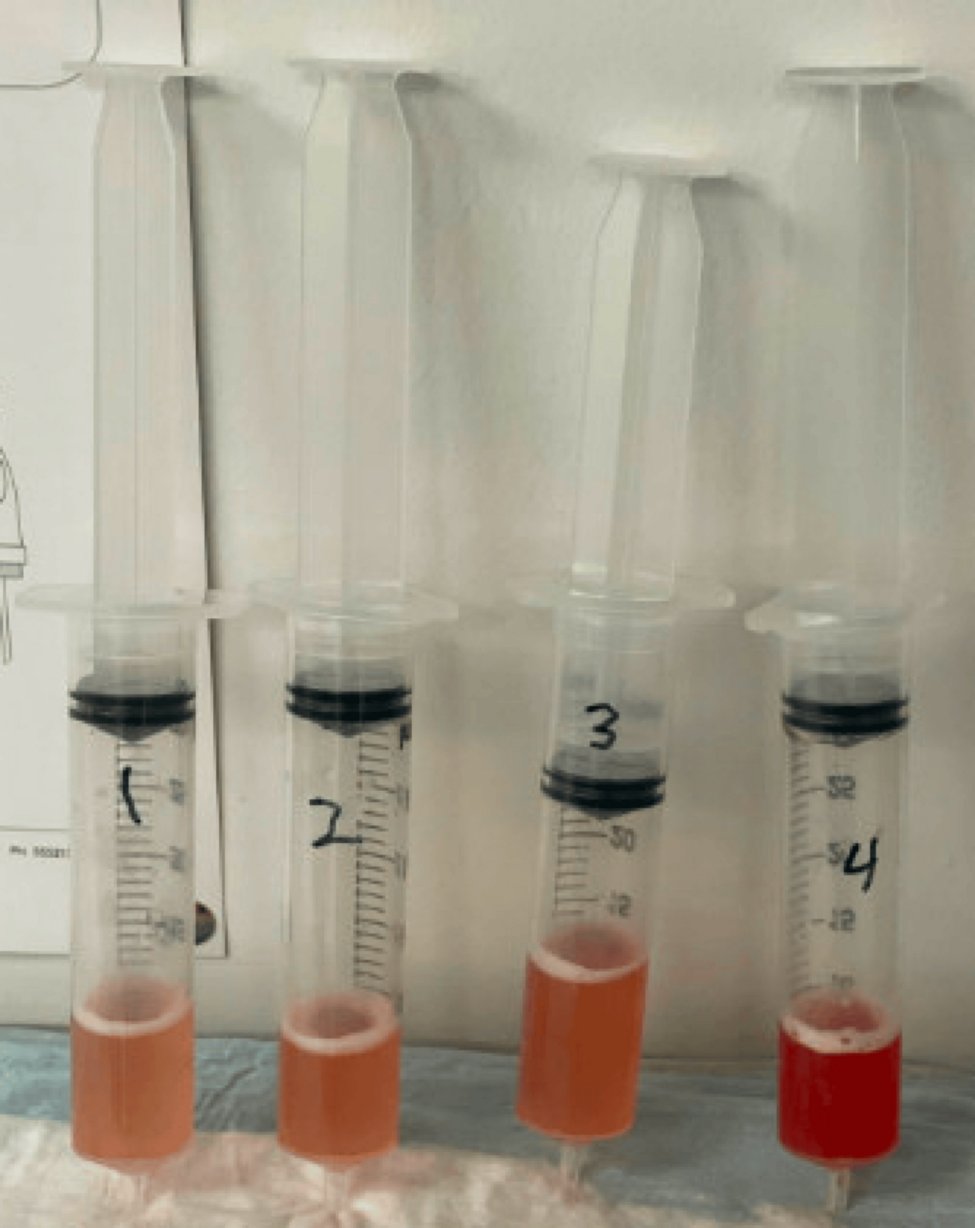
Bloody aliquots collected sequentially were listed as 1 - 4 with bronchoalveolar lavage (BAL) confirming diffuse alveolar hemorrhage (DAH)

The patient continued to have persistent hemoptysis, so pulmonology was consulted. A bronchoscopy found edematous and friable mucosa friable throughout all segments, with no endobronchial lesions or sources of active bleeding visualized. Four aliquots were collected from the right middle lobe, with an increase in the bloody appearance of each sequential aliquot, consistent with the presentation for DAH shown in Figure 3. The bronchoalveolar lavage sample contained greater than 14,000 red blood cells/UL. Further laboratory work was collected with negative antineutrophilic cytoplasmic antibodies (ANCAs), antinuclear antibody (ANA), anti-glomerular basement membrane (anti-GBM), and interferon-gamma release assay (IGRA). Acid-fast bacillus, gram stain, and fungal cultures grew no organisms. Cytology showed benign bronchial cells. The patient’s hemoptysis resolved and she was able to be weaned from supplemental oxygen. She was discharged the following day in a stable condition.

Here, we report a case of a female patient who presented with shortness of breath after developing a seizure. Further investigations excluded cardiogenic etiology and showed critically low phenytoin levels. It improved within 48 hours of supportive care without giving diuretics favoring the diagnosis of NPE as the primary pathology. The goal of our case report is when dealing with similar cases to keep NPE in mind, and hence provide the appropriate management.

## Discussion

Acute pulmonary edema is a frequent cause of dyspnea and is normally categorized as either cardiogenic or noncardiogenic, with the insults being attributed to cardiovascular or pulmonary origins [2]. Despite these two broad buckets, it is imperative to keep NPE, an infrequent form of pulmonary compromise associated with an acute neurological insult, as a differential. The pathophysiology behind NPE is not well understood, but there are two described mechanisms leading to increased capillary permeability causing pulmonary edema. The first is based on the brain producing an adrenergic response leading to vasoconstriction and increased hydrostatic pressure, and the second is centered around increased inflammatory mediators causing endothelial damage [6-8]. Some triggers for NPE identified in the literature include aneurysmal subarachnoid hemorrhage, cranial trauma, spinal cord injury, stroke, epilepsy, and postoperative intracranial injury [2,3,6,7]. In a study by Mahdavi et al., they recognized the lack of data on NPE following seizures and retrospectively analyzed patients admitted for seizures who underwent thoracic CT, which appeared frequently in post-generalized convulsive seizure patients [8].

DAH is an uncommon yet life-threatening condition that is characterized by hemoptysis, diffuse pulmonary infiltrates, and hypoxemic respiratory failure. It is associated with autoimmune conditions such as vasculitis, coagulation disorders, cardiac disorders, pulmonary infections, and toxic exposures [4]. You et al. studied the 13 patients described in the literature who had tonic-clonic seizures before experiencing DAH [9]. They found that all the patients had a negative serologic workup for other causes of DAH and they experienced rapid restoration of oxygenation status with supportive care. This supported the likelihood of the Muller maneuver during a seizure, negative pressure of inspiration against a closed glottis, and inflicted traumatic rupture of the capillaries leading to DAH [9].

For this patient, the proposed mechanism of respiratory compromise and hemoptysis is also seizures causing NPE leading to DAH. Workup that led to NPE-induced DAH includes negative autoimmune panels (ANCA, ANA, antiphospholipid antibodies, anti-GBM), infectious panels (human immunodeficiency virus, hepatitis, respiratory viral panel, blood cultures, urine culture, respiratory culture, IGRA, acid-fast bacillus smear) and unremarkable BAL findings. Normal cardiac function was noted on transthoracic echocardiogram.

Steroids are often prescribed in the management of DAH, however, there are no clear guidelines for the management of DAH secondary to neurologic insults [10]. For our patient, while steroids were considered, the rapid resolution of symptoms with supportive care proved to be sufficient. This supports that the neurological insult from her seizure led to NPE, alveolar damage and subsequent DAH. Had there been another origin for the DAH, supportive care likely would not lead to adequate resuscitation.

The treatment of NPE is based on the resolution of cranial injury and supportive ventilation, with seizure control as the only preventable treatment [7]. NPE can be fatal and the clinical relevance of recognizing NPE early can help to avoid unnecessary treatments since the cornerstone of treatment is supportive care [2,3,6-8].

## Conclusions

NPE typically presents following a neurological insult and can lead to respiratory failure requiring mechanical ventilation. NPE can present with hemoptysis, however, this is a rare presentation. Our case presents a patient following a seizure with respiratory distress in the setting of DAH. The patient rapidly improved with supplemental oxygen and BAL to clear the bilateral lung fields of residual blood. Diagnoses related to infection, autoimmune disorders, cardiogenic causes, toxic insults, and other known etiologies of DAH were excluded. It is important for clinicians to consider NPE on the differential for patients with pulmonary edema following a neurological inciting event in order to provide adequate supportive care and avoid treatments and interventions that have side effects that can incite further harm in patients. Further studies should be considered to study the pathophysiology of NPE and consider more directed therapies.
